# Anti-rheumatic treatment is not associated with reduction of pentraxin 3 in rheumatoid arthritis, psoriatic arthritis and ankylosing spondylitis

**DOI:** 10.1371/journal.pone.0169830

**Published:** 2017-02-22

**Authors:** Gia Deyab, Ingrid Hokstad, Jon Elling Whist, Milada Cvancarova Småstuen, Stefan Agewall, Torstein Lyberg, Barbara Bottazzi, Pier Luigi Meroni, Roberto Leone, Gunnbjorg Hjeltnes, Ivana Hollan

**Affiliations:** 1 Department of Medical Biochemistry, Innlandet Hospital Trust, Lillehammer, Norway; 2 Lillehammer Hospital for Rheumatic Diseases, Lillehammer, Norway; 3 Department of Research, Innlandet Hospital Trust, Brumunddal, Norway; 4 Institution of health care—Health science PhD programme, Oslo and Akershus University College, Oslo, Norway; 5 Oslo University Hospital, Ullevål, Oslo, Norway; 6 Institute of Clinical Sciences, University of Oslo, Oslo, Norway; 7 Department of Medical Biochemistry, Oslo University Hospital, Ullevål, Oslo, Norway; 8 Humanitas Research Hospital, Rozzano, Milan, Italy; 9 Department of Clinical Sciences and Community Health, University of Milan, Milan, Italy; 10 IRCCS Istituto Auxologico Italiano, Milan, Italy; 11 ASST G Pini, Milan, Italy; 12 Department of Medicine, Innlandet Hospital Trust, Lillehammer, Norway; 13 Department of Medicine, Brigham and Women’s Hospital, Boston, United States of America; 14 Harvard Medical School, Boston, United States of America; Keio University, JAPAN

## Abstract

**Background:**

Pentraxin 3 is proposed to be a marker of inflammation and cardiovascular risk, but its role in inflammatory rheumatic diseases (IRDs) is still uncertain. Therefore, we wanted to examine if anti-rheumatic treatment reduced serum PTX3 (s-PTX3) levels in IRDs, and if s-PTX3 levels were related to other markers of inflammation and to endothelial function (EF).

**Methods:**

We examined s-PTX3, EF and established inflammatory biomarkers in 114 IRD patients from the PSARA study before and after 6 weeks and 6 months of treatment with methotrexate (MTX) or anti-tumor necrosis factor alpha (anti-TNF) therapy with or without MTX co-medication.

**Results:**

s-PTX3 levels in all IRD diagnoses were above the upper limit of the reference range. In contrast to established inflammatory markers, in particular CRP and ESR, s-PTX3 levels did not change significantly after 6 weeks and 6 months of anti-rheumatic therapy. There was no difference in change in s-PTX3 levels from baseline to 6 weeks and 6 months between MTX monotherapy and anti-TNF regimens. CRP, ESR and EF were not related to changes in s-PTX3 neither in crude nor adjusted analyses.

**Conclusion:**

IRD patients have increased s-PTX3 levels, which, in contrast to other inflammatory markers, do not seem to improve within 6 months of therapy with MTX and/or anti-TNF. Thus, s-PTX3 might reflect a persisting immune process, even a causal factor of inflammation, not inhibited by the standard anti-rheumatic treatment. Furthermore, even though s-PTX3 is thought to be a strong predictor of cardiovascular prognosis, it was not related to EF.

## Introduction

Patients with inflammatory rheumatic diseases (IRDs) have increased cardiovascular (CV) morbidity and mortality, predominantly due to accelerated atherosclerosis. The reason to premature cardiovascular disease in IRDs has not been fully clarified, but immune dysregulation and inflammation appear to play important roles [1, 2].

Inflammation is known to be involved in the pathogenesis of all stages of the atherothrombotic process, from initiation of endothelial dysfunction (ED), to atheroma formation, plaque destabilization and thrombogenesis [3]. It is well known that increased levels of systemic inflammatory biomarkers, such as C-reactive protein (CRP), predict cardio vascular disease (CVD) development and are related to CVD severity [4]. During the last years, there has been increasing interest in another protein from the pentraxin family (which CRP belongs to), i.e. pentraxin 3 (PTX3). There is evidence suggesting that PTX3 might be at least as good independent predictor of CV risk as CRP [5–7]. In contrast to CRP, which is produced in the liver upon stimulation by interleukin-6 (IL-6), PTX3 is produced directly in the inflamed tissue. Furthermore, it is stored in granules of neutrophils, ready to be rapidly released upon microbial stimulation [8–10]. Thus, the PTX3 response is faster than the CRP response, and PTX3 is thought to more accurately reflect the actual inflammatory situation [11].

PTX3 is produced in the vessel wall in response to pro-inflammatory cytokines such as interleukin-1beta (IL-1β) and tumor necrosis factor alpha (TNF) [12]. For example, PTX3 has been observed in atherosclerotic plaques, and there are theories suggesting that systemic PTX3 levels might be a useful indicator of acute coronary syndrome, because of its reflection of vascular inflammation [3, 13–15].

Similar to CRP, PTX3 is a pattern recognition molecule of the immune system, and has multiple important functions, including anti-microbial effects, participation in clearance of apoptotic cells, and regulation of inflammation [8]. Several studies have reported increased PTX3 levels in IRDs. Some of these studies suggested that PTX3 might be related to the increased CV risk in IRD [8, 16, 17]. However, the real role of PTX3 in inflammation and premature CVD in IRD has not been fully elucidated yet. For instance, it is still unknown how PTX3 responds to anti-rheumatic treatment, and whether it might be used as a biomarker of IRD activity and CV risk.

Therefore, the aim of this study was to examine if anti-rheumatic treatment in form of methotrexate (MTX) and/or anti-TNF (anti-TNF) reduced serum PTX3 (s-PTX3) levels in IRDs, and if s-PTX3 levels were related to other inflammatory markers, and to endothelial function (EF).

## Patients and methods

### Patients

A total of 140 patients, 74 with rheumatoid arthritis (RA), 40 with psoriatic arthritis (PsA) and 26 with ankylosing spondylitis (AS) were enrolled in the PSoriatic arthritis, Ankylosing spondylitis, Rheumatoid Arthritis (PSARA) study at Lillehammer Hospital for Rheumatic Diseases between October 2008 and May 2010. The study was retrospectively registered with the following trial registrations: Clinicaltrials (NCT00902005); The Norwegian Regional Ethical Committee (S-07377b) and the Norwegian Biobank register (2054). Written consents were obtained from all patients included in the study.

Inclusion criteria were as follows: males and females with age range 18–80 years; PsA according to Moll and Wright 1973 criteria [18], AS according to the modified New York diagnostic criteria for ankylosing spondylitis [19] or RA according to the ACR 1987 criteria [20]; clinical indication for starting with either MTX monotherapy or anti-TNF treatment with or without MTX co-medication (anti-TNF±MTX). Women with childbearing potential had to use a reliable method of contraception.

Exclusion criteria included lack of co-operability, any contraindication for MTX and anti-TNF, any significant infection (including subclinical tuberculosis), immunodeficiency, pregnancy or breastfeeding, congestive heart failure, uncontrolled diabetes mellitus, recent stroke (within 3 months), demyelinating disease, use of systemic glucocorticoid > 10 mg/day during the last 2 weeks or anti-TNF during the last 4 weeks before the inclusion, malignancy and any chronic inflammatory disease other than RA, AS or PsA.

The patients were examined at baseline and after 6 weeks and 6 months of treatment. Of all included patients (140), 114 completed the 6 months follow-up. The reasons for dropout were as follows: side-effects in 12 patients, insufficient treatment response in 11 patients, hepatitis C in 1 patient, failure in logistics in 2 patients (patients were not summoned for follow- up).

### Treatment

The type and doses of anti-rheumatic treatment were decided by clinical rheumatologists not involved in the study, upon clinical judgment, and in accordance with the Norwegian guidelines. Doses were as follows: etanercept 50 mg subcutaneous (SC) injection once a week, adalimumab 40 mg SC injection every other week, infliximab 3–5 mg/kg intravenous injection at baseline, then following standard dosing regimen. MTX doses were 15–25 mg orally once a week.

Clinical guidelines consider MTX as first line of anti-rheumatic treatment in RA and some other IRDs, especially in those with peripheral joint arthritis [21]. On the other hand, due to limited effect of conventional disease modifying anti-rheumatic drugs (DMARDs), including MTX, in axial spondyloarthritis (including AS and PsA), TNF inhibition is the treatment of choice in most patients with axial spondylarthritis who do not sufficiently respond to non-steroidal anti- inflammatory drugs (NSAIDs) [22, 23].

### Clinical tests

The data collection included demographic data, medical history, life-style information and medication (including previous and current use of DMARDs and systemic glucocorticosteriods, NSAIDs, statins and other drugs known to affect the cardiovascular system).

At all three visits, EF was assessed by the Reactive Hyperemia Index (RHI) measured by a fingertip plethysmograph (EndoPAT 2000; Itamar). A finger probe were placed on the index fingers of each hand and a blood pressure cuff was placed on the right upper arm, while the other arm functioned as the control arm. The right upper arm was occluded for 5 min and then released. The RHI was calculated as the ratio between the average post-obstructive pulse wave amplitude (PWA) and the average of pre-occlusion PWA. This is described in more details in Hjeltnes et al, and Onkelinx et al [24, 25]. Endothelial dysfunction was defined as RHI <1.67, in accordance with the cut-off level determined for patients at risk for coronary artery disease [26]. Furthermore, the patients were examined by several self-reported and clinical instruments for evaluation of their disease activity and severity adequate for their condition such as Bath Ankylosing Spondylitis Disease Activity Index (BASDAI), Bath Ankylosing Spondylitis Patients Global Score (BAS-G), Bath Ankylosing Spondylitis Metrology Index (BASMI), Bath Ankylosing Spondylitis Functional Index (BASFI), Medical health Assessment Questionnaire (MHAQ), Disease Activity Score for 28 joints (DAS28), Physicians' Global Assessment Score of disease activity (PGA) and Patients' Global Assessment Score of disease activity (PtGA) (Table 1).

**Table 1:** Baseline patient characteristics.

Characteristics	RA (n = 64)	PsA (n = 30)	AS (n = 20)
Age	57 (28–79)	50 (23–78)*	*49 (30–72)****¥***
Rheumatic disease duration, years	2 (0–30)	3 (0–37)	3 (0–40)
Male (gender), n (%)	17 (27)	17 (57)*	*16 (80)****¥***
Current smokers, n (%)	20 (31)	7 (23)	10 (50)
Hypertension, n (%)	17 (27)	7 (23)	6 (30)
Diabetes mellitus, n (%)	3 (5)	0 (0)	1 (5)
Methotrexate, n (%)	34 (53)	16 (53)	*0 (0)****ɸ¥***
Endothelial dysfunction, n (%)	23 (36)	8 (27)	9 (45)
PTX 3 (ng/mL)	3.8 (1.2–22.1)	4.2 (1.1–14.7)	4.0 (1.9–8.5)
CRP (mg/L)	8 (1–78)	5 (1–99)	10 (1–157)
WBC (10^9^/L)	7.25 (3.7–11.3)	6.3 (4.2–11.3)	*7*.*9 (4*.*7–12*.*3)****ɸ***
ESR (mm/h)	18.5 (1–81)	7 (2–48)*	*9*.*5 (2–87)* ***¥***
Total Cholesterol (mmol/L)	5.2 (2.8–8.7)	5.3 (3.8–7.1)	4.9 (2.9–7.9)
LDL (mmol/L)	3.2 (1.1–5.8)	3.4 (2.4–4.9)	2.8 (1.6–5.2)
HDL (mmol/L)	1.4 (0.9–2.8)	1.3 (0.7–2.9)	1.25 (0.8–2.5)
Triglycerides (mmol/L)	1.2 (0.5–2.8)	0.96 (0.6–3)	1.25 (0.7–2.1)
HbA1C (%)	5.7 (4.9–8.9)	5.5 (4.6–6.4)	5.6 (4.9–6.9)
Fasting serum glucose (mmol/L)	5.1 (4.2–8.6)	5.1 (4.4–6.9)	5 (4.5–8.4)
Uric acid μmol/L	271 (151–499)	296 (152–566)	310 (182–410)
BMI (kg/m^2^)	26 (19–41)	26 (19–39)	28 (22–36)
Previously used DMARDs, n (%)	40 (63)	15 (50)	2 (10)
MHAQ	0.65 (0–1.45)	0.40 (0.05–1.55)	0.43 (0–1.40)
PGA ¤	38(7–73)	21 (0–57)*	*26 (3–60)****¥***
PtGA ¤	52 (5–98)	44 (2–96)	56 (6–96)
Number of swollen joints	6 (0–28)	2 (0–8)*	*0 (0–6)****¥ɸ***
BASDAI	-	4.7 (0.3–9.5)	5.1 (0.9–9.6)
BASFI	-	3,1(0–7.2)	4.1 (1.1–7.6)
BASMI	-	-	3 (0–10)
DAS 28	4.98 (2.6–7.3)	-	-

Unless indicated otherwise, values are given as median (range).

RA, rheumatoid arthritis; PsA, psoriatic arthritis; AS, ankylosing spondylitis; PTX3, pentraxin 3; CRP, C-reactive protein; WBC, white blood cells; ESR, erythrocyte sedimentation rate; LDL, low-density lipoprotein; HDL, High-density lipoprotein; HbA1C, glycated haemoglobin; BMI, body mass index; MHAQ, Medical Health Assessment Questionnaire; PGA, Physicians' Global Assessment Score of disease activity; PtGA, Patients' Global Assessment Score of disease activity; BASDAI, Bath Ankylosing Spondylitis Disease Activity Index; BASFI, Bath Ankylosing Spondylitis Functional Index; BASMI, Bath Ankylosing Spondylitis Metrology Index; DAS 28, Disease Activity Score for 28 joints.

* p< 0.01 for comparisons between the RA group and the PsA group.

¥ p< 0.01 for comparisons between the RA group and the AS group.

ɸ p< 0.01 for comparisons between the PsA group and the AS group.

¤ On a 100-mm visual analog scale.

### Blood samples

Blood samples were drawn after fasting for 8 hours (including non-allowance of smoking). Routine hematological and biochemical tests including erythrocyte sedimentation rate (ESR), white blood cells (WBC) and CRP, were performed at all visits using test standards of the local hospital laboratory. Furthermore, small aliquots of serum and plasma were stored at -80°C for later analyses (including PTX3 analysis). PTX3 levels were determined in serum by a homemade sandwich enzyme-linked immunosorbent assay based on the murine monoclonal antibody MNB4 as capturing antibody, and a rabbit antiserum (pAb) raised against human PTX3, affinity purified and biotinylated, as detection antibody [8, 27]. Samples were assessed in batches and in random order, by assessor blinded for clinical data. The procedure was performed as described for human plasma, with the only addition of a preincubation step of serum samples with Polybrene-EDTA. Briefly 1μl Polybrene-EDTA (2.5% polybrene; 2.5% EDTA in phosphate buffered saline without calcium and magnesium, pH 7.4) was added to 50μl of undiluted serum samples and incubated for 10 minutes at room temperature. Human serum samples were then diluted and added to MNB4 coated wells. Absorbance at 450 mm was measured after incubation with pAb and then streptavidin-horse radish peroxidase. Mean PTX3 content was calculated converting Abs450 values to protein concentration by means of a standard curve with recombinant purified human PTX3 (range from 75 pg/ml to 2.4 ng/ml). The assay has a sensitivity of 100 pg/ml and interassay variability ranges from 8% to 10%. No cross-reaction was observed with CRP and serum amyloid P component.

### Statistical analyses

As all continuous variables were not normally distributed, non-parametric tests such as Mann-Whitney U test and Wilcoxon sign test were applied for comparisons between and within the examined groups. Chi-square test was used for comparison of categorical data between the study groups. Linear regression analyses were used to assess associations between s-PTX3 and selected laboratory and clinical variables. The multiple regression models were adjusted for the central variables of interest and for age and gender (as gender and age are known to influence PTX3 levels) and for baseline characteristics that were statistically significantly related to PTX3 in simple regression analysis and in analysis adjusted for age and gender only [7]. We performed two multiple linear regression models: one to investigate if s-PTX3 was independently related to EF (Model I) and one to examine if s-PTX3 was independently related to established inflammatory markers (CRP and ESR) (Model II).

All analyses are considered exploratory; however due to multiple testing, P-values ≤0.01 were considered statistically significant, and all statistical tests were two-sided.

All analyses were performed in IBM SPSS statistics, Version 23.

## Results

### Patient characteristics

Baseline characteristics are described in Table 1. Gender and age in the RA group differed significantly from the AS group and PsA group. AS patients had the highest proportion of patients with ED, and PsA patients the highest level of s-PTX3, but none of these differences were statistically significant. RA patients had significantly higher levels of PGA than PsA and AS patients. Number of swollen joints (NSJ) differed significantly in all patient groups (highest in RA and lowest in AS).

MTX was initiated in about half of the RA and PsA patients, but in none of the AS patients (as a result of guidelines for treatment of these diseases).

### Changes in established markers of disease activity and PTX3 during anti-rheumatic treatment

**Fig 1:**
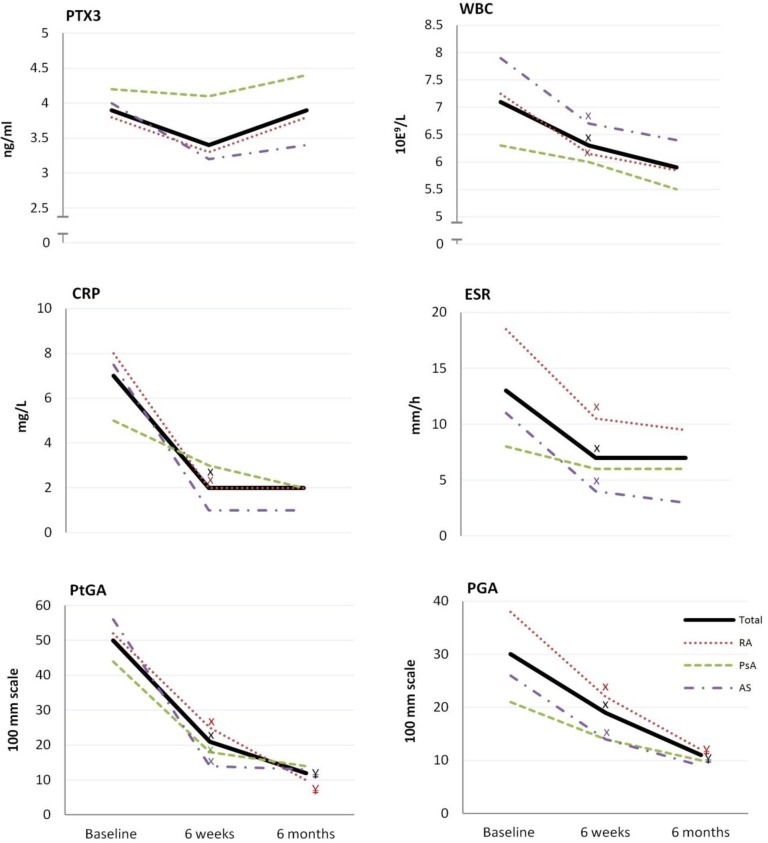
PTX3 and established markers of disease activity at baseline, 6 weeks and 6 months of treatment. Values are given in median. IRD, inflammatory rheumatic disease; RA, rheumatoid arthritis; PsA, psoriatic arthritis; AS, ankylosing spondylitis; PTX3, pentraxin 3; WBC, white blood cells; ESR, erythrocyte sedimentation rate; CRP, C-reactive protein; PGA, Physicians Global Assessment Score of disease activity; PtGA, Patients' Global Assessment Score of disease activity. X p<0.01 for difference between the evaluation at baseline and at 6 weeks ¥ p<0.01 for difference between the evaluation at 6 weeks and 6 months.

Fig 1 shows the changes in s-PTX3, WBC, CRP, ESR, PGA and PtGA at all visits. In the entire patient sample, the median s-PTX3 levels were above the upper limit of the reference range (1–3 ng/ml) at all visits. For the total IRD group and for all the three diagnostic groups, there was a tendency towards decrease in s-PTX3 levels after 6 weeks of treatment, and towards increase from 6 weeks to 6 months of treatment; however, none of these changes reached the level of statistical significance.

In the total IRD group and in the RA group, CRP, ESR, WBC, PGA and PtGA levels decreased significantly after 6 weeks of therapy (p<0.001 for all). Of these variables, only the PGA and PtGA in RA and in the total IRD group (p<0.003 for both) decreased further from 6 weeks to 6 months, while no additional decrease in CRP, WBC and ESR levels was observed in any of the examined groups. In the PsA group, no statistically significant changes in any of the examined markers were revealed, except for the PtGA (Fig 1). The AS group had a statistically significant decrease in all of the examined markers from baseline to 6 weeks, except for CRP and s-PTX3 (Fig 1).

### Effects of MTX monotherapy and anti-TNF±MTX treatment on s-PTX3

There were no statistically significant changes in the s-PTX3 levels between patients treated with MTX and anti-TNF±MTX in the total IRD group and in the RA and PsA groups (not shown).

As all AS patients were treated with anti-TNF, no comparison was possible between the two treatment regimens in this group.

### Relationship of other clinical and laboratory factors to PTX3

In univariate regression analyses, there were no associations between s-PTX3 and CRP, ESR, RHI, PGA, PtGA, NSJ, and WBC (Table 2).

**Table 2:** Predictors of PTX3.

	Unadjusted analyses			Adjusted analyses			Adjusted analyses		
				Model 1			Model 2		
	B	95% CI	p-value	B	95% CI	p-value	B	95% CI	p-value
Age	-0.033	-0.070 to 0.005	0.090	-0.037	-0.075 to 0.002	0.062	-0.032	-0.073 to 0.009	0.126
Gender	0.656	-0.187 to 1.500	0.126	0.789	-0.078 to 1.656	0.074	1.086	0.180 to 1.992	*0*.*019*
CRP	0.004	-0.017 to 0.025	0.709				0.023	-0.005 to 0.052	0.109
ESR	-0.013	-0.038 to 0.012	0.305				-0.037	-0.074 to 0.000	0.048
WBC	-0.003	-0.271 to 0.265	0.982						
RHI	-0.231	-1.230 to 0.768	0.647	-0.070	-1.064 to 0.925	0.890			
PGA	0.014	-0.011 to 0.039	0.270						
PtGA	-0.005	-0.023 to 0.013	0.575						
NSJ	0.145	-0.075 to 0.365	0.192						

Predictors of PTX3. CRP, C-reactive protein; ESR, erythrocyte sedimentation rate; RHI, reactive hyperaemic index; PGA, Physician's global assessment of disease activity; PtGA, Patient's global assessment of disease activity; NSJ, number of swollen joints.

Model 1; R^2^ = 0.048, Model 2; R^2^ = 0.054.

Furthermore, s-PTX3 was not related to hemoglobin, thrombocyte count and neutrophil count, IRD duration or CV risk factors, neither in crude analyses nor in analyses adjusted for age and gender and inflammatory markers. There were no statistically significant associations between s-PTX3 and DAS28 for RA patients, and BASDAI and BASFI for AS and PsA patients, in neither univariate analyses nor analyses adjusted for age and gender.

Model I: Neither there were significant relationships between s-PTX3 and RHI after adjustments only for age and gender. RHI was not related to s-PTX3 in analyses adjusted for age, gender, IRD and traditional CV risk factors (smoking, BMI, hypertension, hyperlipidemia and hypercholesterolemia). Similar results were obtained when we evaluated EF in terms of ED (dichotomous variable) instead of RHI (continuous variable).

Model II: Neither there were significant relationships between s-PTX3 and ESR and CRP in analyses adjusted only for age and gender. s-PTX3 was not statistically significantly related to neither CRP nor ESR in analyses adjusted for age, gender and ESR and CRP.

IRD was not related to s-PTX3 in any of the multiple regression models.

## Discussion

The main findings in this novel study were as follows: 1) s-PTX3 levels did not change significantly with anti-rheumatic treatment, in contrast to other inflammatory markers and clinical disease activity measures. 2) There was no difference in the effect of MTX monotherapy and anti-TNF ±MTX treatment (in MTX failures) on s-PTX3 levels in RA and PsA patients. 3) s-PTX3 was not statistically significantly related to other systemic inflammatory markers. 4) s-PTX3 was not statistically significantly related to EF.

The reference range of s-PTX3 applied by the laboratory was 1–3 ng/mL [7]. The median s-PTX3 baseline value in the studied IRD group was 3.9 ng/mL, and it stayed above the upper limit at all visits. Other studies have shown that PTX3 levels are higher in several IRDs (RA, PsA, AS, polymyalgia rheumatica, giant cell arteritis, systemic lupus erythematosus and small vessel vasculitis) compared to control groups [8, 16, 17, 28, 29]. A recent systematic review also confirmed that both serum and plasma levels of PTX3 in autoimmune diseases were significantly higher than in normal controls [30].

However the s-PTX3 values in the present study were different from those observed in other studies. Compared to the IRD patients in the study of Hollan et al. (PTX3 mean = 2.1 ng/ml), our patients had higher s-PTX3 values, even though the s-PTX3 analyses were performed in the same laboratory, using the same method [8]. This might possibly be attributed to a higher disease activity in the current patient group, as all patients, recruited from a rheumatology clinic, were in need of initiation of anti-rheumatic treatment due to active IRD, while the IRD patients in Hollan et al. previous study were recruited with connection to their coronary artery bypass surgery, during a period of a relatively low disease activity.

Compared to Okan et al., who measured PTX3 values in PsA (median = 11.21 ng/ml), our patients had lower s-PTX3 levels [16]. These differences might most likely be due to different laboratory techniques, because also the control group had higher PTX3 values than the reference range in our laboratory.

The median s-PTX3 levels in our patient population were comparable to the patients with small vessel vasculitis (mean = 3.24 ng/ml) in the study by Fazzini et al. [29].

There were no statistically significant differences in s-PTX3 at baseline between RA, PsA and AS patients (Fig 1), despite the differences in their demographic characteristics and measures of IRD disease activity, including ESR and PGA (Table 1).

These differences might be accidental, but they may also be due to a real difference in these systemic inflammatory markers at the point of time when these patients were in need of initiation or intensification of their anti-rheumatic therapy.

Several markers of disease activity (CRP, ESR, WBC, PGA and PtGA) decreased rapidly after 6 weeks and 6 months of therapy indicating reduction of inflammation, while there was no statistically significant change in s-PTX3 levels in the total IRD group, nor in the different diagnostic groups after 6 weeks and 6 months of anti-rheumatic treatment.

The PsA group had the highest s-PTX3 levels at all visits, and at 6 weeks, CRP, ESR, WBC and PGA levels had not decreased significantly, in contrast to the RA and AS group (except for CRP in AS group; see limitations. Thus, inflammation, in terms of these parameters appeared to be less susceptible to MTX or anti-TNF±MTX treatment in PsA than in RA and AS [31]. This remains to be examined in further studies.

Notably, s-PTX3 levels were not positively related to traditional markers of disease activity, such as CRP and ESR, at baseline. Furthermore, the changes in s-PTX3 between baseline and 6 weeks and 6 months visits were not related to changes in CRP and ESR during the same period of time. Our findings are in accordance with previous studies that did not find any positive correlation between PTX3 and other inflammatory markers including CRP, ESR and WBC in patients with PsA, small vessel vasculitis, Takayasu’s arteritis, acute myocardial infarction patients and AS [17, 29, 32–34]. However, although PTX3 does not seem to be related to CRP in psoriasis, it is reportedly positively related to the extent and severity of skin psoriasis [35].

In a study of PsA patients, their PTX3 baseline levels did not differ from healthy controls.

However, after 24 months of anti-TNF treatment, the PTX3 levels significantly increased within the PsA group compared to the baseline[36].

Taken together, our results indicate that s-PTX3 does not reflect the actual systemic disease activity in RA, PsA and AS patients, and disease activity amelioration after anti-rheumatic treatment, determined by the traditional clinical and biochemical measures of disease activity.

However, it is important to keep in mind that the anti-rheumatic treatment does not target the cause of these IRDs, and that it usually does not lead to a total and permanent remission of the disease activity (characterized by a total absence of any inflammatory activity).

Thus, in hypothesis, s-PTX3 might reflect an active inflammatory pathway that sustains PTX3 production even in patients apparently responding to the traditional anti-rheumatic therapy. Hence, further studies are needed to explore if PTX3 might be a marker of the residual inflammatory activity in patients with apparently satisfying therapeutic response.

It is not clear what are the principal sources and triggers of the increased PTX3 levels in IRDs. PTX3 might originate from various cells involved in local inflammatory processes, e.g. from circulating neutrophils, in joints and in the cardiovascular system. In theory, the residual production of PTX3 in IRD might occur in vessels, as there are indications that inhibition of inflammatory disease activity does not prevent progression of vascular damage in PsA [37].

Further studies are needed to clarify the roles of different possible triggers for the increased PTX3 production, such as autoimmune inflammation, infections (including latent infection due to IRD-related dysregulation or immunosuppressive therapy), and atherogenic lipoproteins [12, 13, 38, 39].

Based on our results, we cannot say with certainty what is the reason to persistent high PTX3 levels in patients with apparent low disease activity as determined by the common measures, such as CRP. However, one might speculate that the local/neutrophilic PTX3 production may persists, e.g. due to persistent stimulation by certain triggers or by reduced inhibition of the PTX3 response, even in patients with low levels of systemic inflammation (not sufficient to induce high production of CRP in the liver and marked inflammatory symptomatology). It is possible that PTX3 mirrors an underlying pathology of IRD, which is not well-reflected by the established disease activity markers, such as CRP. Hence, PTX3 might have an advantage in reflecting subtle pathologic, and even pathogenetic, changes in IRD compared to CRP. Though, this hypothesis has to be tested in further studies as our study is not designed to give an answer to this question.

PTX3 is thought to be a strong predictor of CV risk. Consequently, we expected to find a positive association between s-PTX3 and EF [6–8]. To our knowledge, the association between PTX3 and EF has not been examined before. Our data do not support the notion that PTX3 might be a good biomarker for ED and the associated CV risk. A hypothetical explanation for the lack of association between s-PTX3 and EF, could be that damaged endothelial cells increase and maintain their PTX3 production. According to Bjorklund et al., endothelial cells from irradiated human artery express PTX3 even years after the irradiation [40]. In theory, this could suggest that damage to endothelial cells may result in prolonged PTX3 induction, perhaps to protect the vasculature. However, as mentioned above, PTX3 may also stem from other cells and tissues, such as from inflamed joints.

MTX and anti-TNF inhibit inflammatory activity through different modes of action [41, 42]. TNF is one of the key pro-inflammatory cytokines involved in the pathogenesis of RA and other IRDs, and TNF inhibition represents one of the most efficient and common types of current anti-rheumatic therapy [41, 43]. MTX, a folate-antagonist, is an anchor anti-rheumatic drug given as the drug of choice to most patients with newly detected chronic peripheral arthritis. It appears to reduce disease activity by multiple actions, such as by inhibition of secretion of IL-1β and other pro-inflammatory molecules [42]. Thus, one might speculate that the high s-PTX3 levels in our IRD patients with active disease might be partly secondary to their high levels of pro-inflammatory cytokines, e.g., TNF and IL-1β. However, as neither TNF-inhibition nor MTX treatment seemed to reduce s-PTX3 levels, other factors than these cytokines are likely to significantly contribute to the excess PTX3 formation and secretion in our patient sample [44].

Because PTX3 is an important molecule of the innate immunity response, protecting against pathogens, its increase might even reflect an underlying, currently unknown, cause of IRDs, such as an ongoing infection. Thus, there is a need to examine in further studies how PTX3 levels influence long-term outcomes in IRD, and what it is induced by.

In theory, the high CV risk associated with high PTX3 might be secondary to the trigger of PTX3 production and release, rather than to PTX3 itself (i.e., PTX3 might be just a bystander of this relationship). It is possible that the trigger of PTX3 expression might be involved in the pathogenesis of CVD and/or IRD [27].

In fact, in animal models, PTX3 administration has been observed to protect from CVD, probably via modulation of the complement cascade and immuno-inflammatory balance and other mechanisms [45, 46]. Hence, it is possible that the maintenance of high PTX3 production in IRDs might be beneficial, counteracting negative effects of the traditional and non-traditional CV risk factors [45, 46].

### Limitations

Our study is burdened by common disadvantages of an observational study, such as differences in baseline characteristics between the groups as the patients were not randomly selected. However, it has been increasingly recognized that observational studies also possess advantages compared to randomized control trials, e.g., increased reproducibility due to a real-life population, better safe-guarding of ethical principles as they allow for providing optimal individualized treatment for the patients, etc. [47].

To compensate for baseline differences between the groups, we adjusted for several baseline characteristics in multiple regression models.

It is important to keep in mind that there are essential differences between IRD patients starting with MTX monotherapy and those starting with anti-TNF. For example, as MTX is the drug of choice in most patients with peripheral chronic arthritis, patients with these conditions who receive anti-TNF treatment are likely to have longer disease duration, and a more severe disease, more refractory to anti-rheumatic therapy (Table 1).

We cannot evaluate differences in monotherapy with MTX or anti-TNF as most of the patients using anti-TNF also used MTX co-medication. Nevertheless, all the PsA and RA patients using anti-TNF were MTX-failures, i.e. they did not get a sufficient effect of MTX prior to the initiation of anti-TNF treatment. Thus, it is likely that the MTX effect on disease activity in the IRD group is relatively poor, and that the MTX is provided first of all to reduce side-effects of anti-TNF therapy.

The p-value was set to 0.01, to decrease the risk of Type-one error (false positive findings). On the other hand, this approach increases the chance of Type-two error (false negative findings). For example, the lack of significant decrease in CRP in the PsA and AS group during the treatment is likely to be due to this phenomenon (as the findings would be significant at 5% level of significance).

A great advantage of our novel study is a well-characterized study population, and design that makes it possible to compare the effect of two of the main anti-rheumatic treatment regimens on s-PTX3 in three common IRDs.

## Conclusion

In conclusion, our data revealed that anti-rheumatic treatment with MTX and TNF±MTX did not affect the increased s-PTX3 levels in IRD patients. s-PTX3 was not related to any established markers of disease activity. It is therefore possible that s-PTX3 might reflect a persisting immune process, even a causal factor of the inflammation, not inhibited by the standard anti-rheumatic treatment.

Furthermore our data do not support the notion that s-PTX3 might be a good biomarker of CV risk in IRD as it was not related to EF.

## Supporting information

S1 File SPSS file. SPSS file containing all data underlying the statistical analysis performed in this study.
(SAV)
